# The Nrf1 CNC-bZIP Protein Is Regulated by the Proteasome and Activated by Hypoxia

**DOI:** 10.1371/journal.pone.0029167

**Published:** 2011-12-21

**Authors:** Nikolai L. Chepelev, Joshua D. Bennitz, Ting Huang, Skye McBride, William G. Willmore

**Affiliations:** 1 Department of Biology, Carleton University, Ottawa, Ontario, Canada; 2 Institute of Biochemistry, Carleton University, Ottawa, Ontario, Canada; Instituto de Química - Universidade de São Paulo, Brazil

## Abstract

**Background:**

Nrf1 (nuclear factor-erythroid 2 p45 subunit-related factor 1) is a transcription factor mediating cellular responses to xenobiotic and pro-oxidant stress. Nrf1 regulates the transcription of many stress-related genes through the electrophile response elements (EpREs) located in their promoter regions. Despite its potential importance in human health, the mechanisms controlling Nrf1 have not been addressed fully.

**Principal Findings:**

We found that proteasomal inhibitors MG-132 and clasto-lactacystin-β-lactone stabilized the protein expression of full-length Nrf1 in both COS7 and WFF2002 cells. Concomitantly, proteasomal inhibition decreased the expression of a smaller, N-terminal Nrf1 fragment, with an approximate molecular weight of 23 kDa. The EpRE-luciferase reporter assays revealed that proteasomal inhibition markedly inhibited the Nrf1 transactivational activity. These results support earlier hypotheses that the 26 S proteasome processes Nrf1 into its active form by removing its inhibitory N-terminal domain anchoring Nrf1 to the endoplasmic reticulum. Immunoprecipitation demonstrated that Nrf1 is ubiquitinated and that proteasomal inhibition increased the degree of Nrf1 ubiquitination. Furthermore, Nrf1 protein had a half-life of approximately 5 hours in COS7 cells. In contrast, hypoxia (1% O_2_) significantly increased the luciferase reporter activity of exogenous Nrf1 protein, while decreasing the protein expression of p65, a shorter form of Nrf1, known to act as a repressor of EpRE-controlled gene expression. Finally, the protein phosphatase inhibitor okadaic acid activated Nrf1 reporter activity, while the latter was repressed by the PKC inhibitor staurosporine.

**Conclusions:**

Collectively, our data suggests that Nrf1 is controlled by several post-translational mechanisms, including ubiquitination, proteolytic processing and proteasomal-mediated degradation as well as by its phosphorylation status.

## Introduction

Nrf1 (nuclear factor-erythroid 2 p45 subunit-related factor 1) belongs to the cap-n-collar (CNC) subfamily of basic leucine zipper (bZIP) transcriptional factors including Nrf1, Nrf2, Nrf3, p45NFE2 (p45NFE2, nuclear factor-erythroid 2 p45 subunit), Bach1 (BTB (Broad-complex, Tramtrack, and Bric-a-brac) and CNC (cap'n'collar) homology 1, basic leucine zipper transcription factor) and Bach2. These factors must bind to small Maf or c-Jun proteins prior to DNA binding [1]. The sequence required for DNA-binding of CNC-bZIP (cap'n'collar/basic leucine zipper) factors is known as the electrophile response element (EpRE; also referred to as the antioxidant response element (ARE)) with a consensus sequence of 5′-TGAnnnnGC-3′ [2]. Antioxidant and cytoprotective genes, regulated transcriptionally through their EpREs, include NAD(P)H:quinone oxidoreductase 1, the glutathione-S-transferases, ferritin, heme oxygenase-1, catalase and superoxide dismutase [3]. Since Nrf1 controls phase 2 detoxification enzymes that aid in metabolism and removal of potential carcinogens, and due to the fact that potent EpRE-inducers such as sulforaphane are known chemopreventive agents, understanding the mechanisms of Nrf1 regulation may aid in the development of cancer therapeutics. Furthermore, due to the cytoprotective nature of phase 2 enzymes against oxidative stress-induced neurodegeneration [4], manipulation of the upstream factors controlling these enzymes (e.g., Nrf1 and Nrf2) could be useful in the search of therapeutic targets against chronic neurodegenerative diseases [4].

Currently, the mechanisms controlling Nrf2 activity have been studied in great detail, while studies examining Nrf1 regulation are lacking. However, it has been shown that Nrf1 may play as an important role in human pathologies as Nrf2 [5]. Nrf2 was initially thought to be kept in the cytosol under homeostatic conditions by interaction with Keap1 (Kelch-like ECH (erythroid cell-derived protein with cap'n'collar homology)-associated protein 1) [6], which favors its rapid ubiquitination and degradation by the proteasome [7]. When the cell encounters oxidative stress, the Keap1-mediated proteasomal degradation of Nrf2 is compromised [8], allowing Nrf2 to dissociate from Keap1 and translocate to the nucleus to activate EpRE-driven gene expression. Further analysis has shown that homeostatic Keap1-Nrf2 interactions are not permanent and take place in the nucleus *via* transient shuttling of Keap1 into that compartment [9]. In contrast, Nrf1 is not regulated by Keap1 [10]. Instead, the activity of Nrf1 appears to be negatively controlled by its N-terminal domain (NTD), which directs Nrf1 to the endoplasmic reticulum (ER) [10]–[12]. Nrf1, but not Nrf2 or Nrf3, is essential for embryonic development; *nrf1* ^−/−^ mice die at mid-late gestation, presumably due to anemia-induced hypoxia [13]. Recently a unique set of genes, controlled by Nrf1, have been identified and contain metallothioneins-1 and -2 (MT1 and MT2 respectively) [14].

**Figure 1:**
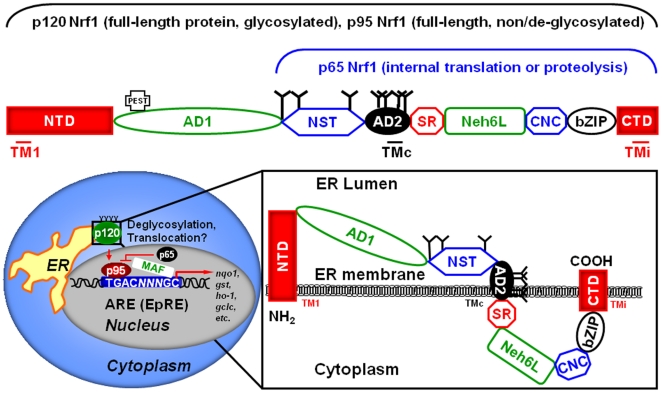
The structural domains of Nrf1, its function and predicted topology within the ER membrane. The structural domains in human Nrf1 (hNrf1) amino acid sequence were identified using a multiple amino acid alignment with mouse Nrf1 (mNrf1) sequence and domain designations as reported in [10]. The hNrf1 domains are: NTD, N-terminal domain (amino acids 1–124); AD1, acidic domain 1 (amino acids 125–324); NST (Asn/Ser/Thr-rich region) (amino acids 325–432); AD2, acidic domain 2 (amino acids 433–482); SR (Ser repeat) domain (amino acids 483–519); Neh6L (Neh (Nrf2-ECH homology) 6-like) domain (amino acids 520–611); CNC, cap'n'collar domain (amino acids 612–655); bZIP, basic Leu zipper domain (amino acids 656–717); CTD, C-terminal domain (amino acids 718–772). The topology of mNrf1 was predicted by Zhang and others [11]. TM1, TMi and TMc are putatitve trans-ER membrane regions. Nrf1 is synthesized as an ER-targeted protein and, once inserted into the ER membrane *via* TM1, TMi and TMc, is glycosylated in the ER lumen; the Nrf1 glycoprotein is referred to as p120. Following the translocation of the luminal part of the p120 into the nucleoplasm, it is deglycosylated to become active p95 Nrf1, heterodimerizes with small Maf or c-Jun proteins and binds to the EpRE to activate the expression of genes involved the antioxidant defense and phase II detoxification metabolism. Internal translation or proteolysis gives rise to p65 Nrf1, dominant negative repressor of the EpRE-driven gene expression. Glycosylation sites are represented by “Y”. Drawing of the domains and specific regions of Nrf1 were performed to scale.

Nrf1 localizes primarily to the ER (see Figure 1) as well as the nuclear envelope membrane [11]. The ER membrane-resident form of Nrf1 represents a low activity, glycosylated protein with an apparent molecular weight of 120 kDa (p120), while the nuclear form (p95) is an active, non-glycosylated (or deglycosylated) protein [15, see also Figure 1]. The ER location of Nrf1 is thought to be suitable for the maintenance of the ER redox homeostasis [11], perhaps by affecting the ER membrane lipid organization *via* its amphipathic, transmembrane α-helices and participating in membrane-dependent biological events [16]. It has also been proposed that the localization of Nrf1 within the ER determines the activity of this CNC factor and that the ER redox status and Nrf1 glycosylation status could cause Nrf1 to relocate from the ER to the nucleus [11]. As ER-resident Nrf1 is entirely glycosylated and nuclear-localized Nrf1 is entirely non- or deglycosylated, it has been hypothesized that Nrf1 deglycosylation could represent the main mechanism of its regulation [11]. Also, it has been hypothesized that Nrf1 is activated by the proteasomal cleavage of its N-terminus to remove its inhibitory NTD, producing smaller, more active forms of Nrf1 [17]. In addition, there is a p65 form of Nrf1, presumably arising from Nrf1 translation initiating at an internal start codon [18] although the possibility of the p65 arising as a result of a proteolytic cleavage can not be ruled out [15]. p65 has been shown to act as a dominant negative inhibitor of Nrf2-mediated, EpRE-driven luciferase activity [19]. Apart from antagonistic competition of Nrf1 p65 (and, potentially, full-length Nrf1) with Nrf2 for the EpRE binding site, the *nrf2* promoter contains two EpRE sequences [20], which may provide Nrf1 with yet another means of regulating Nrf2 expression.

Recent studies have established Nrf1 as a pivotal transcriptional regulator of the genes of subunits of the proteasome. Thus, Nrf1 activates proteasome gene expression upon proteasome inhibition treatment in human Ea.hy926 cells [21] and mouse embryonic fibroblasts [22] to compensate for the loss of proteasome activity. Similarly, Nrf1 conditional knock-out studies in mice brain lead to proteasomal impairment, further confirming the importance of Nrf1 as a translational regulator of the proteasomal gene expression [23]. Although previous work provided solid evidence for the link between Nrf1 and proteasome, the relationship between different forms of Nrf1 has been essentially absent or received very little attention in the previous studies. Here, we present the evidence supporting two levels of proteasomal regulation of Nrf1 function. In addition, we describe the hypoxic inducibility of Nrf1 and potential regulation of Nrf1 function by phosphorylation. The present study shows that exogenous Nrf1 activity correlates inversely with the abundance of the p65 form of Nrf1, a known inhibitor of the EpRE-mediated gene expression. The derepression of Nrf1 activity by the removal of the inhibitory p65 Nrf1 form may be one of several determinants of Nrf1 activation under hypoxia.

## Results

### Proteasomal inhibition stabilizes p120 and ubiquitinated Nrf1

**Figure 2:**
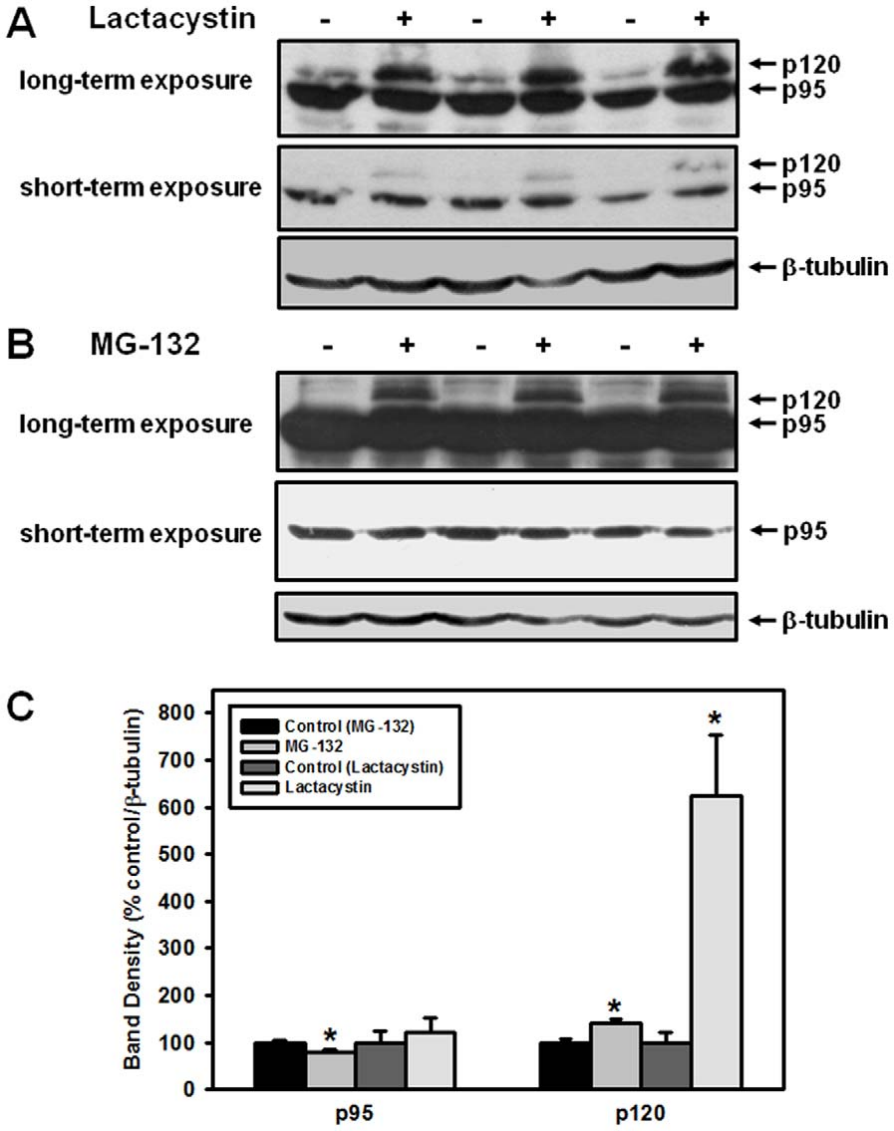
Nrf1 is stabilized by proteasomal inhibition in COS7 cells. COS7 cells were treated with 5 µM lactacystin (**A**) or 10 µM MG-132 (**B**) for six hours and Western blots were performed with anti-Nrf1 antibody as described in the Materials and Methods section. DMSO (the solvent used for lactacystin and MG-132) was used for controls. (**C**) Shown is the densitometry analysis of the blots using the results of four independent experiments (means ± SEM). Asterisks (*) indicate significant difference (p<0.05) compared to control.

Closely-related CNC-bZIP factors Nrf2 and Nrf3 have been shown to be controlled through keeping their intracellular levels low under homeostatic conditions through the proteasomal degradation [24], [25]. To test if Nrf1 is also regulated *via* proteasomal degradation, we analyzed its protein expression in the presence of proteasomal inhibitors clasto-lactacystin-β-lactone (lactacystin) and MG-132. Proteasomal inhibition with both inhibitors greatly enhanced the expression of inactive, ER-bound glycosylated form of Nrf1, p120 (Figures 2A and B). Interestingly, while the density of the p120 band increased significantly (about 140% compared to controls) during MG-132 treatment, the intensity of the band, corresponding to the active, nuclear glycosylated/deglycosylated p95 Nrf1 form decreased about 20% compared to DMSO (Figures 2B and C). This could imply that higher levels of p120 could arise, at least in part, due to increased glycosylation of p95, in response to proteasomal inhibition. Noteworthy is that the decrease in the level of p95 observed in Figure 2B is subtle (20% only) and, while being statistically significant, may not be biologically relevant. In addition, no alteration of the p95 expression was not seen for lactacystin as the p95 expression during this treatment was unaltered, indicating that the stabilization of p120 may proceed independently of p95 Nrf1. When COS7 cells were treated with an oxidative stressor 2,2′-azobis(2-amidinopropane) dihydrochloride (AAPH), we observed the accumulation of p95 form without any apparent effect on the p120 form of Nrf1 (see Figure S1). This result suggests that there may be some time lag between the accumulation of p120 and p95 forms of Nrf1, given the time required for the deglycosylation conversion of p120 to p95 Nrf1.

### Nrf1 contains a PEST motif and is rapidly degraded

**Figure 3:**
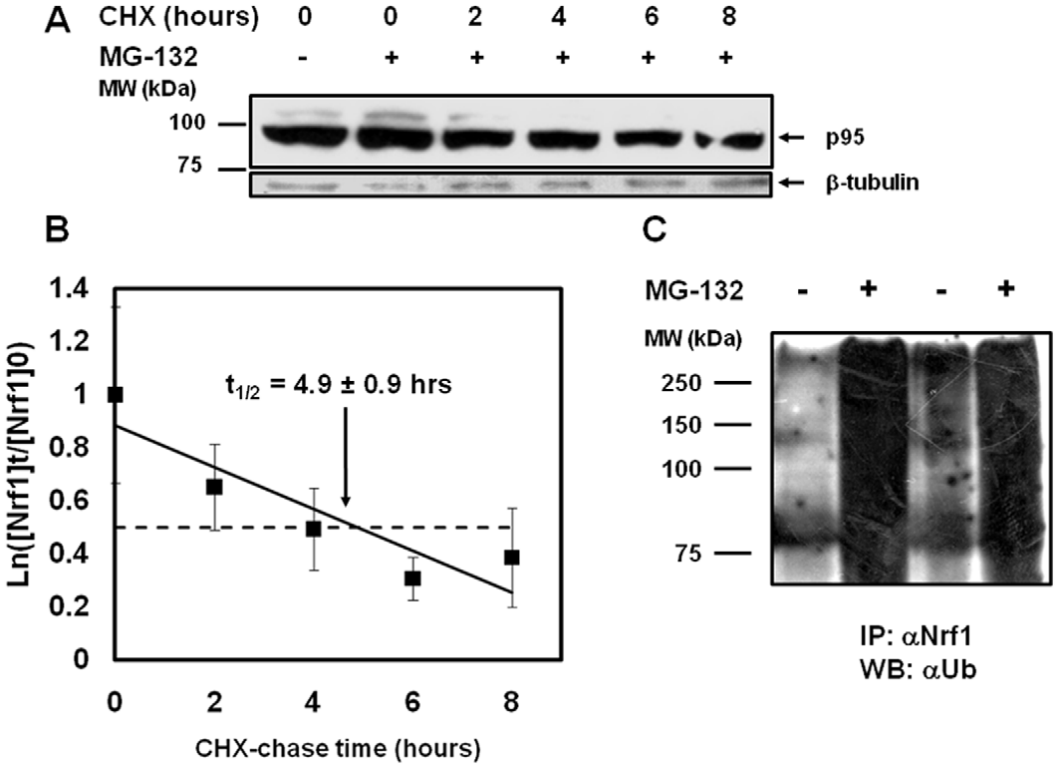
Nrf1 is a short-lived protein and undergoes ubiquitination and proteasomal degradation. (**A**) COS7 cells were treated with 12 µM MG-132 for five hours, the medium was replaced by fresh medium with 100 µg/mL CHX and the cells were harvested at different time intervals (0–8 hours) and subjected to immunoblotting for Nrf1 p95. (**B**) The graphical determination of Nrf1 p95 half-life (t _1/2_) using at least three independent experiments is shown. (**C**) Immunoprecipitation was performed as described above in the Materials and Methods section on the lysates of COS7 cells pre-treated with 10 µM MG-132 or DMSO for six hours and the results of two independent experiments are shown.

Nrf2 and Nrf3 have half-lives (t_1/2_) of approximately 15 and 30 minutes, respectively [24], [25] and contain two strong putative PEST sequences [25], [26]. PEST sequences are rich in Pro, Glu, Thr and Ser and are found frequently in rapidly degraded proteins; the Ser and Thr residues are potential phosphorylation sites [27]. We used the PESTfind analysis tool (http://www.at.embnet.org/toolbox/pestfind/) to search for any putative PEST sequence on Nrf1 and found that Nrf1 has a strong PEST motif (amino acids 141–169) in its NTD (see Figure 1 for Nrf1 domains). To test whether Nrf1 is indeed subject to rapid degradation, as suggested by the presence of strong PEST motif, we applied cycloheximide (CHX) and analyzed Nrf1 protein degradation by immunoblotting when protein synthesis was inhibited (Figure 3A). A plot of band density versus time (Figure 3B) showed that the t_1/2_ of p95 and p120 is approximately 5 hours (data not shown). Compared to transcription factors Nrf2 and Nrf3, Nrf1 is more than 5 to 10 times more stable, but the Nrf proteins are less stable than other proteins that have t_1/2_ between 16 (lysozyme) and 210 (phosphoglycerate kinase) hours [28]. Nrf1 can therefore be regarded as a relatively short-lived protein; however, relatively long half-life, compared to that of other transcription factors, suggests that Nrf1 activity may be regulated by other mechanisms, such as post-translational modifications. Next, we found that anti-Nrf1 antibody was able capture significantly higher levels of ubiquitinated Nrf1 protein, in co-immunoprecipitation experiments, as revealed by immunoblotting with anti-ubiquitin antibody (Figure 3C). Thus, Nrf1 is precisely controlled by keeping its levels low through its continuous ubiquitination and degradation by the proteasome. Given its relatively long half-life, compared to Nrf2 and Nrf3, Nrf1 may have additional modifications that allow for ubiquitination, similar to the large subunit of RNA polymerase II (RPB1), which requires phosphorylation prior to ubiquitination in order to be degraded by the proteasome [29]. Ubiquitination of the target protein would be dependent upon conditions required for the primary post-translational modification to occur. Thus turnover of Nrf1 may be higher under conditions that favour other post-translational modifications.

In addition to ubiquitination, we noticed the disappearance of a high molecular weight form of Nrf1 with an approximate molecular weight of 250 kDa (p250) as a result of CHX treatment, oxidative stress (AAPH or tert-butylhydroquinone (tBHQ)) and hypoxia treatments (see Figure S2). Furthermore, we observed a similar decrease in the p250 from content in aged mice tissues (data not shown). The identity of this stress-modulated band merits further investigations and could represent post-translational modification of Nrf1 (e.g., ubiquitination), Nrf1 homodimer, ER membrane-bound Nrf1 or Nrf1 tightly bound to an unknown protein.

### Comparison of Nrf1 protein expression under proteasomal inhibition and hypoxia

With the observation that MG-132 and lactacystin stabilized p120, we wanted to determine if the protein expression of Nrf1 is also affected by hypoxia. To this end, we subjected COS7 and WFF2002 cells to hypoxic conditions (1% O_2_). Hypoxia is known to induce the expression of genes involved in iron metabolism, many of which are transcriptionally regulated *via* the CNC-bZIP factors to which Nrf1 belongs [30]. Similarly, hypoxia activates the expression of MT1 and MT2 [31]; these genes contain the EpRE sequence and are known targets of Nrf1, but not Nrf2 [14]. Intracellularly, hypoxia treatment has many similarities with proteasomal inhibition; for instance, hypoxia-inducible factor alpha subunits (HIFαs) are stabilized when the proteasome is inhibited, which leads to HIFα-mediated activation of hypoxia-inducible gene expression. In addition, multiple studies have reported an increased rate of reactive oxygen species (ROS) production under hypoxia [32] and ROS are known activators of the EpRE-Nrf1 pathway. Given that hypoxia results in the generation of ROS, we wanted to study the effects of hypoxia on Nrf1 function in order to elucidate the mechanisms of Nrf1 regulation in greater detail.

**Figure 4:**
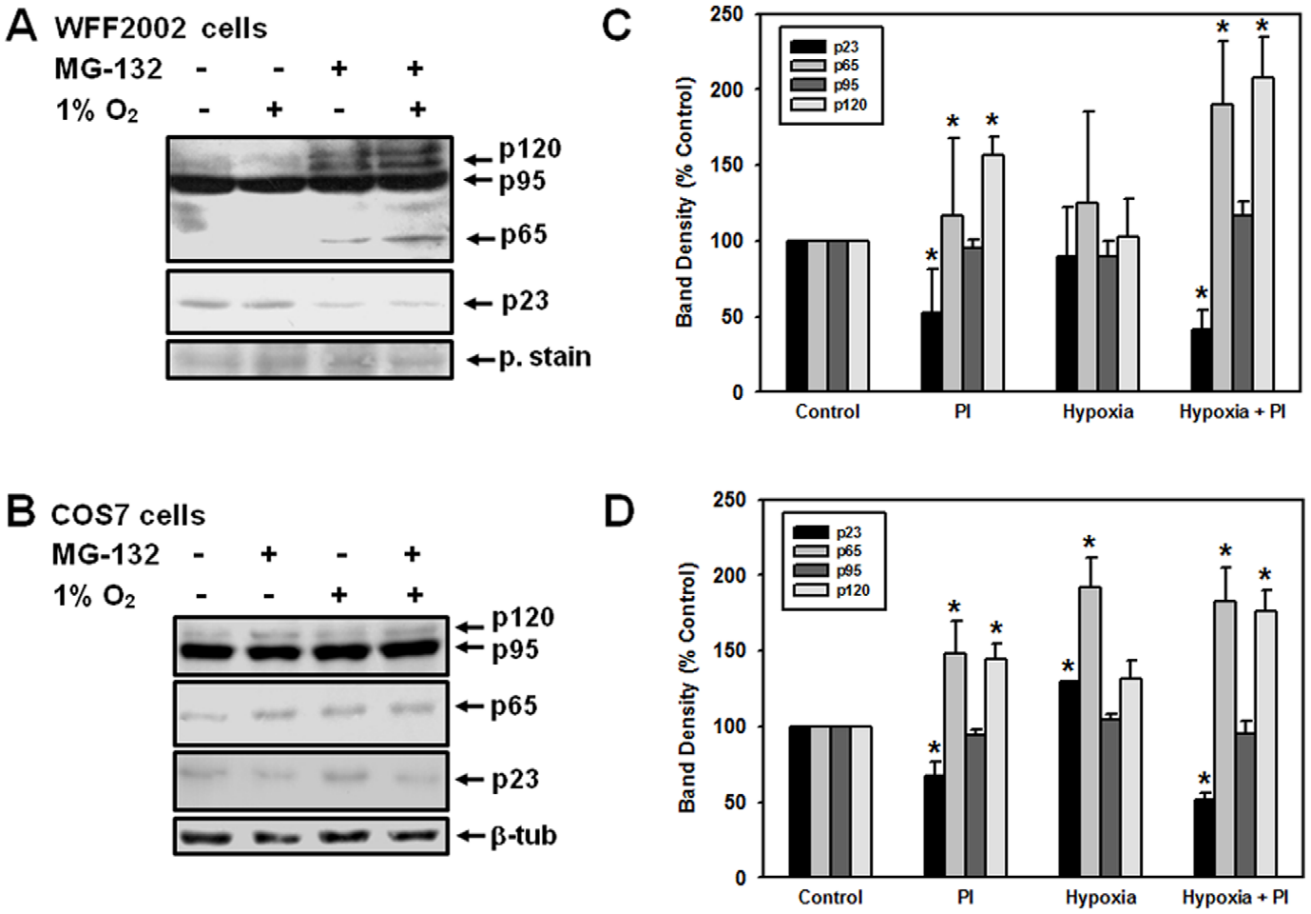
Comparison of the Nrf1 expression under hypoxia and proteasomal inhibition. WFF2002 (**A**) and COS7 (**B**) cells were treated with normoxia (21% O_2_), hypoxia (1% O_2_), 10 µM MG-132 or hypoxia and MG-132 combination for six hours and subjected to Western blotting. The band density was normalized with respect to β-tubulin (β-tub) or ponceau S red staining (p. stain) and is presented as means ± SEM of at least three independent experiments for WFF2002 (**C**) and COS7 (**D**) cells. Asterisks (*) indicate significant difference (p<0.05) compared to control.

Unlike MG-132 treatment, no stabilization of p120 form was observed in hypoxic treatments for both cell lines (Figures 4A and B), suggesting that the effect of combining these two treatments is not different from applying each treatment individually. Since the accumulation of p120 form was also noticeable in WFF2002 cells (Figure 4A), the mechanism of Nrf1 control through proteasomal degradation is not likely to be cell-specific. The band density corresponding to p65 Nrf1, a dominant negative inhibitor of EpRE-driven gene expression [19], was markedly increased after 6 hours of MG-132 treatment in WFF2002 cells (Figure 4A) and in both MG-132- and hypoxia-treated COS7 cells (Figure 4B). Very little is currently known about p65 and its role in EpRE-mediated gene expression, apart from its negative effect on the EpRE pathway and cell type-specific accumulation of p65 might provide some hints towards its function and regulation. We observed no changes at the mRNA levels for all four conditions tested (data not shown), supporting the involvement of post-translational modifications in Nrf1 regulation by the proteasome and hypoxia.

Interestingly, in both cell lines, the intensity of the band migrating as a 23 kDa protein (“p23” Nrf1) was diminished during proteasomal inhibition (see Figures 4A and B). Since our anti-Nrf1 antibody recognizes the N-terminal fragment of Nrf1 (amino acids 191–475), and p23 is always highly expressed, this suggests that this is a product of N-terminal cleavage, in addition to the ubiquitin-dependent proteasomal degradation discussed above. p23 likely represents either protease- and/or a stable, proteasome-generated N-terminal fragment of Nrf1. This is in line with the previous studies, suggesting that p120 Nrf1 must be processed into smaller forms in order to remove the inhibitory NTD and allow the protein to act as a transcription factor (see [17] and references therein). The N-terminal cleavage of Nrf1 might be an important way of regulating this factor as that would allow it to escape ER and to translocate to the nucleus, where it could activate the expression of the EpRE-controlled genes. Other ER-bound transcriptional factors are similarly regulated, such as the sterol-regulatory-element-binding proteins SREBP1 and SREBP2, as well as activating transcription factor 6 (ATF6; *via* intramembrane proteolysis), and this regulation has been well-characterized (see [12] and references therein). Previous studies [15], [16] failed to map any proteolytic cleavage site within the first 170 amino acids at the N-terminus; however, the studies were performed at normal homeostatic conditions and the possibility of Nrf1 regulation by intramembrane proteolysis under stimulated conditions cannot be ruled out [16]. In contrast, a more recent study [21] provided some, albeit indirect, support for the intramembrane proteolysis of Nrf1 prior to its translocation to the nucleus from the ER.

### MG-132 and hypoxia enhances protein binding to EpRE

**Figure 5:**
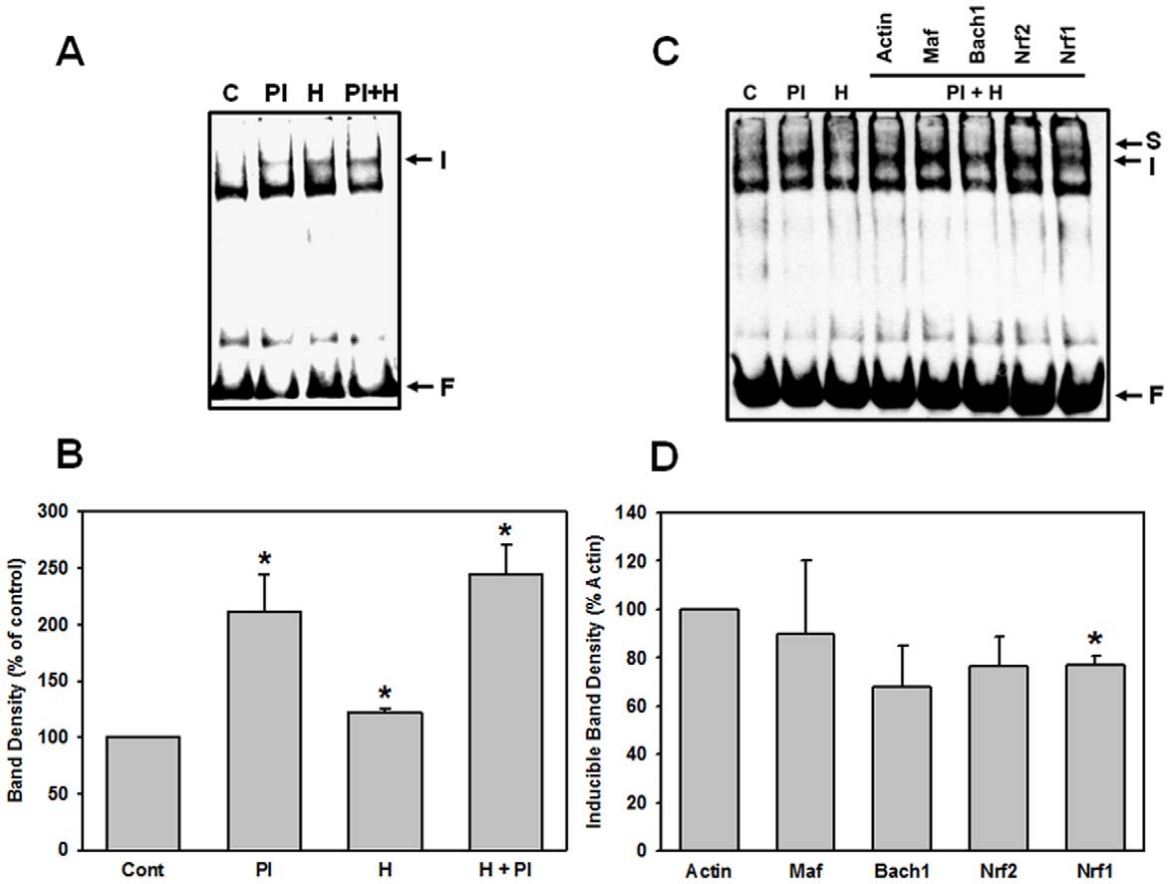
Hypoxia and proteasomal inhibition increase protein binding to the EpRE. Biotin-labelled EpRE probe from *gclm* promoter was applied to 20 µg of total COS7 cell lysate protein in an EMSA format as described in the Materials and Methods section. (**A**) Depicts the appearance of the hypoxia- and MG-132-inducible band. C, PI, H and PI+H designate control (DMSO), proteasomal inhibition (MG-132), hypoxia (1% O_2_ and DMSO) and the combination of hypoxia and MG-132 treatments, respectively. I = induced band, F = free probe. (**C**) The identity of the EpRE-bound complex was probed using antibodies against the proteins indicated in the immunodepletion format. I = induced band, F = free probe, S = supershift. (**B**) and (**D**) are graphical representations of the outcomes of the experiments presented in (**A**) and (**C**), respectively, as the means of band densitometry ± SEM for at least three independent experiments. Asterisks (*) indicate significant difference (p<0.05) compared to control.

Once we showed that proteasomal inhibition stabilizes Nrf1, we investigated whether MG-132 and/or hypoxia have any effects on Nrf1 DNA-binding to the EpRE. Using electrophoretic mobility shift assays (EMSAs), we observed the appearance of MG-132- and hypoxia-inducible bands (Figure 5A), whose density was significantly different from controls (Figure 5B). These results are consistent with the previously reported study of Waleh and co-workers [33], showing hypoxia-inducible DNA-binding to EpREs in human HepG2 and mouse Hepa cells.

To identify the proteins responsible for the hypoxia- and MG-132-inducible EpRE-binding, we used an immunodepletion approach [34]. In this method, an antibody raised against a DNA-binding protein diminishes the intensity of an EMSA band if the DNA-binding protein is, indeed, bound to the target DNA sequence under investigation [34]. Apart from Nrf1, the obvious candidate factors that could be involved in the MG-132- and hypoxia-inducible EpRE complex formation could be Nrf2 as it is stabilized by proteasomal inhibition and Bach1, whose activation by hypoxia has been previously described [35]. As can be seen from Figures 5C and D, anti-Nrf1 antibody significantly diminished the intensity of the inducible band compared to the actin (non-specific) antibody, used as a negative control (Figure 5D). This suggests potential involvement of Nrf1 in the hypoxia- and MG-132-inducible binding to an EpRE probe. However, since enhanced EpRE binding could result in either activation or repression of the EpRE target genes, depending on the nature of the binding factor involved (activator or repressor), we used a luciferase reporter assay, in combination with Nrf1 overexpression, to gain a better understanding of the Nrf1 transactivation function in response to hypoxia and proteasomal inhibition treatments.

### Hypoxia activates while proteasomal inhibition inhibits exogenous Nrf1 activity

**Figure 6:**
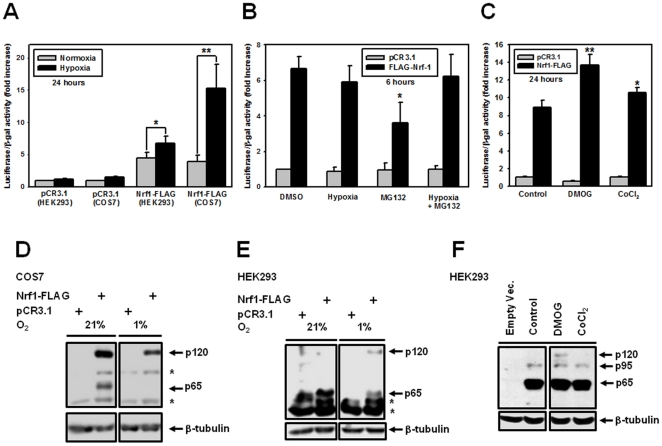
Hypoxia and hypoxic mimetics activate Nrf1, while MG-132 inhibits its transactivation activity. COS7 and HEK293A cells were co-transfected with 3×EpRE-luc, β-galactosidase and Nrf1-FLAG plasmids, treated with hypoxia, 10 µM MG-132, 2.5 mM DMOG or 200 µM CoCl_2_ for twenty-four ((**A**) and (**C**)) or six (**B**) hours as indicated and the luciferase activity in the lysates was measured. The pCR3.1 plasmid, lacking Nrf1, was used as negative control and β-galactosidase activity was used as the transfection efficiency control. The means of at least three independent experiments ± SEM, normalized to the β-galactosidase activity and pCR3.1 are presented. Asterisks (*, **) indicate significant difference (p<0.05 or p<0.001, respectively) compared to control. The lysates of hypoxia-treated (1% O_2_, 24 hours) COS7 (**D**) and HEK293A (**E**) and (**F**) cells were also subjected to immunoblotting using anti-FLAG antibody. In (**D**) and (**E**), asterisks (*) denote the non-specific, cross-reacting bands. Representative blots of three independent experiments are shown.

Next, we tested if proteasomal inhibition and hypoxia have any effect on the transactivation activity of Nrf1. To this end, we used a luciferase reporter vector under the control of three EpREs from chicken β-globin enhancer (3xEpRE-luciferase [21]). Transient co-transfection of 3xEpRE-luciferase with Nrf1-FLAG in COS7 cells showed that 24-hour hypoxia treatments greatly increased the activity of Nrf1-FLAG (Figure 6A). Furthermore, hypoxic activation of Nrf1 is probably not cell type-specific as the same result was seen in HEK293A cells (Figure 6A). In addition to low oxygen, Nrf1-FLAG activation is achievable by hypoxia mimetics cobalt chloride and dimethyloxalylglycine (DMOG, Figure 6C). In contrast, MG-132 markedly decreased the activity of Nrf1-FLAG while hypoxia had no effect on the Nrf1 activity during six-hour treatments (Figure 6B). Again, the action of MG-132 on exogenous Nrf1 activity can be understood in light of the current hypothesis that the processing of Nrf1 by the 26S proteasome could remove its inhibitory NTD (see Figure 1 for Nrf1 domains), targeting Nrf1 to the ER. Blocking Nrf1 processing into p23 and other active forms of Nrf1 (such as p95) by proteasome inhibition would interfere with Nrf1 function, which is in accord with the observed repression of the Nrf1 activity by MG-132 treatment (Figure 6B). Therefore, proteasomal processing seems to be a prerequisite for Nrf1 activation and function.

### Potential effect of p65 on the hypoxia-inducibility of Nrf1

The p65 form of Nrf1 is thought to be produced by either internal proteolysis [15] or as a result of translation initiation from an alternative Met codon [18]. The p65 form of Nrf1 acts as a dominant negative inhibitor of the EpRE-driven gene expression [19] as it contains a DNA-binding domain, but lacks a transactivation domain, such that p65 Nrf1 competes with Nrf2 for the EpRE binding site. As seen in Figures 6D and E, the expression of the p65 form of Nrf1 markedly decreased during hypoxia treatment in both COS7 and HEK293A cell lines, while no other common change was noticeable during hypoxia. Our data presented here is in agreement with the model in which the EpRE-mediated gene expression is activated as a result of down-regulation of the inhibitory p65 form of Nrf1. The two internal Met residues that are thought to give rise to p65 are M321 and M326 [18] which contain consensus Kozak sequences, not present in the M1 codon. Mutating M321 and M326 to M321L and M326L, respectively, resulted in significant loss of basal Nrf1-FLAG activity and hypoxia-inducibility; however, unexpectedly, mutants were not expressed at detectable levels (data not shown). This may be due to increased instability or aggregation of mutant forms of Nrf1 (that are potentially cytotoxic) compared to wildtype Nrf1.

### Nrf1 is activated by phosphorylation

**Figure 7:**
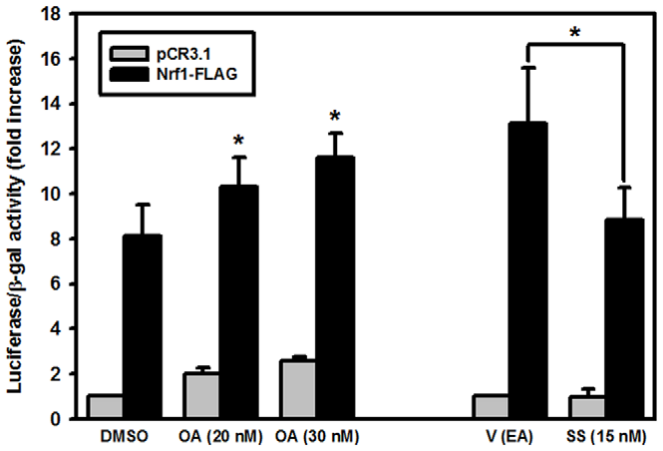
Phosphorylation activates Nrf1 transactivation activity. Luciferase assays were performed on the COS7 cell lysates, co-transfected with 3×EpRE-luciferase, and Nrf1-FLAG plasmids treated with okadaic acid (OA) and staurosporine (SS), to inhibit protein dephosphorylation and phosphorylation, respectively. DMSO or ethyl acetate (EA) was used as vehicle (V) controls in and the concentration of each compound used. The means of at least three independent experiments ± SEM are shown. Asterisks (*) indicate significant difference (p<0.05).

Our computational analyses predicted a high probability of Nrf1 phosphorylation by cdc2 and protein kinase C (PKC) members, especially at the Ser-rich region (NST), facing the ER lumen. Zhang and others [11] provided some evidence that phosphorylation of Nrf1 at its NTD (which contains two potential Tyr phosphorylation motifs, amino acids 62–70 in human Nrf1), may weaken the Nrf1 association with the ER, stimulating the Nrf1 trafficking to the nucleus. Given that phosphorylation by atypical PKC is known to activate Nrf2 [36], we thought that Nrf1 could also be a subject to this modification. To test that, we performed luciferase assays on the COS7 cells co-transfected with Nrf1-FLAG and 3xEpRE-luciferase and subjected to protein phosphatase inhibitor okadaic acid (OA) [24] or pan-PKC inhibitor staurosporine [37]. Figure 7 illustrates that OA activated Nrf1, while staurosporine repressed Nrf1 activity, suggesting that Nrf1 phosphorylation plays a role in Nrf1 transactivation. Notably, the OA treatment resulted in about 2-fold increase of the empty vector-mediated EpRE-luciferase activation, changing the EpRE-driven luciferase activity from 1.0 to 2.0-fold with respect to control vector. This suggests that there is about 1-fold increase in the “noise” signal due to other transcriptional factors, such as Nrf2, being activated through phosphorylation. On the other hand, treatment with OA changed the EpRE-luciferase reporter activity due to Nrf1-FLAG from 8.15 to 10.3-fold with respect to the control. Therefore, the net increase in Nrf1-FLAG activity was 2.15-fold compared to 1-fold increase due to “noise”. These results, as well as a similar study on the effect of OA on Nrf2 (where an approximate 0.5-fold increase in the Nrf2 activity due to OA treatment above background was interpreted as a stimulatory effect of OA on Nrf2 activity (see Figure 6 in [24])), suggests that Nrf1 positively responds to phosphorylation stimulus. Decreased transactivation activity of Nrf1, as a result of staurosporine treatment, strengthens the claim that the phosphorylation status of Nrf1 is important for Nrf1 activity. It is possible that some upstream factors in the signaling cascade that control Nrf1 function responds to phosphorylation/dephosphorylation rather than Nrf1 itself and further studies are required to confirm the target of phosphorylation in the Nrf1 pathway.

## Discussion

### The two levels of the proteasomal control of Nrf1

The levels of key inducible transcription factors are normally kept low under homeostatic conditions through their ubiquitination and continuous degradation to avoid aberrant gene expression. This is the case for transcription factors such as HIFα and the CNC-bZip factors Nrf2 and Nrf3. As expected, we found that Nrf1 was also negatively regulated by the proteasome, being subject to ubiquitination and proteasomal degradation in agreement with Zhang and co-workers, who suggested that Nrf1 abundance may be controlled by the proteasome [11]. We observed high expression of the p23 fragment of Nrf1 under untreated conditions, which diminished following MG-132 treatment. Since p23 would be expected to contain about 210 amino acids, and the recognition region of our antibody is between amino acids 191–475, we believe that p23 represents an N-terminal fragment of the Nrf1 cleaved twice in the amino acids 191–475 region with the distance between the two cleavage sites being approximately 210 amino acids. Further support of this hypothesis has been shown in previous experiments, where Site-1 or Site-2 protease cleavage sites within the first 170 amino acids of Nrf1 could not be identified [15], [16]. Thus, our results support the hypothesis of Zhang and co-workers [15] that Nrf1 is proteolytically cleaved at regions other than NTD. Cleavage by Site-1 and Site-2 intramembrane proteases is a well-known mechanism, responsible for the release of ER-bound transcriptional factors, but the possibility that Nrf1 is cleaved at AD1, NST and AD2 domains merits further investigation. This possibility is also supported by Steffen and co-workers [21], who noticed that the molecular weight of the deglycosylated Nrf1 is still higher than that of the nuclear form of Nrf1 and concluded that intramembrane proteolysis is a feasible mechanism for Nrf1 release to the nucleus from the ER.

The second or “positive” level of Nrf1 regulation by the proteasome, in addition to ubiquitin-mediated proteasomal degradation, appears to be proteolytic processing. In accord with the current hypothesis of the Nrf1 regulation by removal of the NTD domain from full-length Nrf1 [17], proteolytic cleavage of Nrf1 allows it to bypass insertion into the ER and travel to the nucleus. This hypothesis was supported by the fact that proteasomal inhibition with MG-132 not only stabilized p120 Nrf1 and decreased p23 levels, but also repressed Nrf1 activity. A very similar mode of activation, through the processing of p105 to the DNA-binding p50 form of NF-κB by the 26 S proteasome, has been described [38], [39]. A recent study has demonstrated the involvement of Nrf1 in the “bounce-back” response, an elevated proteasomal subunit synthesis which is observed upon proteasome inhibition [22]. According to the study, Nrf1 directly activates the proteasome recovery pathway upon proteasome inhibition. For that to happen, Nrf1 itself must be activated by 1 µM MG-132 treatment, which was shown by the investigators [22]. This is in contrast to our results, as well as the results of others (see Figure 2 of ref. [11]), demonstrating that MG-132 and another proteasome inhibitor, ALLN, applied at 10 or 13 µM concentrations respectively, represses exogenous Nrf1 activity. While both 1 and 10–13 µM concentrations of the proteasome inhibitors were able to stabilize p120 Nrf1, abolishing the proteasomal degradation of Nrf1 completely, it is conceivable that higher concentrations of proteasomal inhibitors are required for the inhibition of the Nrf1 partial processing/activation by the proteasome (which is known to possess several catalytic activities). Thus at low concentrations of proteasome inhibitor, degradation of p120 Nrf1 is inhibited, but not its processing (which activates Nrf1). At high concentrations of proteasome inhibitor, both p120 Nrf1 degradation and processing is inhibited, inhibiting Nrf1 function despite the fact that more Nrf is present. Another possible explanation for this discrepancy is the fact that reporter plasmids with EpREs from different genes were used in previous and our studies and while one set of EpRE-controlled genes can be turned on by a given conditions, other EpRE-driven genes can be turned off due to the complex interplay between co-activator and co-repressor proteins [40].

### Stress-modulated Nrf1 forms

Our findings have emphasized the role of multiple forms of Nrf1. Indeed, Nrf1 is known to exist in several forms, including p120 (glycosylated Nrf1), p95 (non- or deglycosylated Nrf1), inhibitory p65 form as well as 46 and 30 kDa forms [11]. In addition to these, we report the existence of the p23 Nrf1 form, that seems to be a fragment of proteasomal processing of the full-length Nrf1 and another, high molecular weight p250 Nrf1 form, which is destabilized by oxidative stressors, inhibition of protein synthesis and hypoxia. Since the p250 form responds to multiple stimuli (see Figure S2), its characterization will be useful in further attempts to uncover the mechanisms responsible for Nrf1 activation. It is plausible that the p250 form of Nrf1 represents a covalently linked protein-protein or protein-membrane interaction with Nrf1. The other possibility is a currently uncharacterized post-translationally modified form of Nrf1 such as ubiquitinated Nrf1.

### Hypoxic activation of Nrf1 is accompanied by p65 Nrf1 down-regulation

The major significance of our study is that it is the first one to report the alternation in the p65 form expression of Nrf1 in response to a physiologically-relevant condition, hypoxia. The decreased presence of the p65 form of Nrf1, affected by hypoxia, correlated with the activation of Nrf1 activity in accordance with the current knowledge of the inhibitory function of p65 [19]. This is also the first study to report Nrf1 activation by hypoxia. As it has been speculated that the location of Nrf1 in the ER membrane is suitable for the maintenance of the homeostatic redox status of the ER [11], the finding that Nrf1 is a hypoxia-modulated factor is not surprising, given that hypoxia is known to cause the ER stress due to the accumulation of the unfolded proteins in the ER lumen [41]. Among the genes controlled exclusively by Nrf1 (and not Nrf2) are the metallothioneins (MT 1 and 2) [14]. It was found that MT expression is hypoxia-inducible in PCa cells [31] and, according to our study, we think that Nrf1 hypoxic inducibility could be responsible for MT upregulation in response to hypoxia. Nrf1 is remarkable as its full-length p95 form can act as an activator of the EpRE-driven gene expression while its shorter form, p65, acts as an apparent inhibitor of the EpRE pathway. Using transient overexpression of Nrf1-FLAG, we noticed that the levels of the p65 Nrf1 were significantly lowered by hypoxia (Figures 6D and E), which correlated to the increased tranactivation activity of Nrf1-FLAG (Figure 6A), suggesting that the cells possess the ability to control Nrf1 activity by removal of the inhibitory p65 form. However, hypoxic mimetic DMOG was capable of activating Nrf1-FLAG without any apparent effect on the p65 expression (Figures 6C and F), but with pronounced increase of the full-length Nrf1 (p120) expression. This is reminiscent of the recently reported, arsenite-mediated induction of Nrf1, which was characterized by the stabilization of the p120 form of Nrf1 during the treatment of keratinocytes with arsenite [42]. The involvement of other mechanisms such as phosphorylation in the hypoxic inducibility of Nrf1 can further augment activation of this CNC-bZIP factor.

### Concluding comments

**Figure 8:**
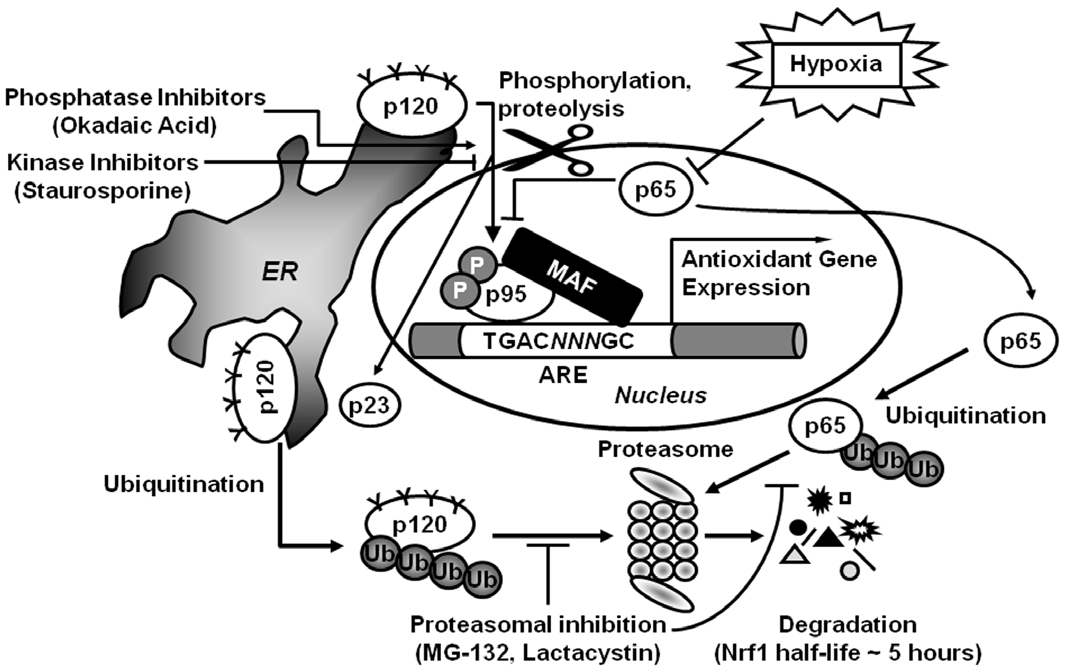
The proposed working model for the Nrf1 regulation by the proteasome and O_2_. Nrf1 is controlled at the level of protein degradation by ubiquitin (Ub)-mediated proteasomal destruction such that the half-life of Nrf1 is approximately five hours. In addition, Nrf1 is also a subject of proteasome-mediated proteolysis during which its N-terminus containing the inhibitory NTD is cleaved to convert Nrf1 from inactive, ER-bound p120 form to the nuclear, active p95 form. Nrf1 can be also activated through its phosphorylation. Hypoxia acts on p65 Nrf1 to diminish its expression. This relieves the Nrf1 p65-mediated repression (by the removal of p65) on the p95 transactivation activity, which, in combination with p95 activation by phosphorylation may greatly affect the expression of the Nrf1-EpRE target genes.

This study has opened up new avenues of research regarding the regulation of Nrf1. What is currently unknown is the kinase and the Nrf1 residue(s) involved in Nrf1 phosphorylation. Zhang and co-workers [11] speculated that Nrf1 phosphorylation can take place at Tyr65 and Tyr77, which could weaken Nrf1 affinity for the ER membrane and stimulate its nuclear import. As well, the proteolytic processing of Nrf1 requires further investigation to identify the protease responsible for as well as the site of the cleavage. Furthermore, it is currently unknown how phosphorylation and processing of Nrf are controlled and if the two processes are interdependent. Given that the p250 form of Nrf1 responds to various stimuli, it would be extremely informative to determine whether this form represents a hyperglycosylated or otherwise modified form of Nrf1, why this form is destabilized under stimulated conditions and what is the significance of that. Our current working model of Nrf1 regulation is summarized in Figure 8. According to our model, Nrf1 is a subject to several regulatory events, including: i) negative regulation by the proteasome through ubiquitin-mediated degradation; ii) positive regulation by the proteasome through proteolytic processing to generate the p23 fragment; iii) phosphorylation and iv) derepression of its transactivation activity by the removal of the inhibitory p65 Nrf1 form.

## Materials and Methods

### Cell Culture, Transfection and Chemicals

HEK293A, COS7 and WFF2002 cells were purchased from the American Type Culture Collection (ATCC, Mannasas, VA). Dubecco's modified Eagle's medium (DMEM), penicillin-streptomycin-antimycotic (P/S/A), horse serum (HS), newborn calf serum (NCS), foetal calf serum (FCS), Opti-MEM I Reduced-Serum Medium and Lipofectamine 2000 were purchased from Invitrogen (Carlesbad, CA). Restriction enzymes and buffers were from New England BioLabs (Ipswich, MA). Okadaic acid, staurosporine and luciferin were from BioShop Canada (Burlington, ON). Complete protease inhibitor cocktail tablets and chlorophenol red-β-D-galactopyranoside (CPRG) were from Roche Diagnostics (Basel, Switzerland). The protein determination assay kit was from BioRad (Hercules, CA). Anti-human Nrf1 antibody (sc-28379) and protein A/G Sepharose beads were purchased from Santa Cruz Biotechnologies (Santa Cruz, CA). Proteasome inhibitors MG-132 and clasto-lactacystin-β-lactone (lactacystin), dimethylsulfoxide (DMSO), phenylmethylsulfonyl fluoride (PMSF), dithiothreitol (DTT), cycloheximide (CHX), 2,2′-azobis(2-amidinopropane) dihydrochloride (AAPH) and other basic chemicals were from Sigma-Aldrich (St. Louis, MO).

### Expression Constructs

A luciferase reporter plasmid containing three EpREs from chicken β-globin enhancer (3×EpRE-luciferase) was a kind gift from Dr. Masayuki Yamamoto (Tohuku University) and has been described elsewhere [43]. Human Nrf1 gene from mammalian gene collection (accession number BC010623) was inserted into a modified pCR3.1 mammalian expression vector using NdeI and EcoRI and then introduced into CMV-5a-FLAG vector (Sigma-Aldrich) using EcoRI and Kpn1 restriction enzymes to create N- and C-terminally FLAG-tagged Nrf1, respectively.

### Cell Culture, Transient Transfection and Luciferase Reporter Assays

COS7 cells were grown in DMEM supplemented with 10% NCS and 3% P/S/A (300 units/mL penicillin G, sodium salt, 300 µg/mL streptomycin sulfate and 0.75 µg/mL Fungizone ® in 0.85% saline) in a humidified atmosphere with 5% CO_2_ at 37°C. HEK293A and WFF2002 cells were grown under the same conditions, but with different sera (10% HS and 10% FCS, respectively). Trypan blue exclusion tests were performed to make sure that the treatments of the cells described here, including hypoxia and proteasomal inhibitor treatments, were not cytotoxic (data not shown). Hypoxic conditions were achieved by setting O_2_ at 1%, CO_2_ at 5% and the balance N_2_ in triple-gas incubators (Thermo Forma, Rockford, IL). Cells were seeded in 6-cm or 6-well plates at a density of 140,000 cells/mL and approximately 24 hours later were transiently transfected with Lipofectamine 2000 as per the manufacturer's protocol (upon becoming at least 70% confluent). For a 6-cm plate, 0.2 µg of β-galactosidase, 8.0 µg of 3×EpRE-luciferase and 2.4 µg of Nrf1-FLAG, or pCR3.1 (empty vector) plasmid DNA, were used. Twenty-four or forty-two hours later, cells were treated for 24 or 6 hours, respectively, and harvested such that the total amount of time after transfection was 48 h. A firefly luciferase reporter gene assay was performed to measure EpRE-driven transcriptional activity. Cells were lysed in 25 to 50 µL of lysis buffer (25 mM glycylglycine (pH 7.8), 1% Triton X-100, 15 mM KH_2_PO_4_, 15 mM MgSO_4_, 4 mM EDTA, 1 mM DTT and a complete protease inhibitor cocktail tablet (Roche)). Approximately 4 to 12 µL of supernatant was added to 76 µL of luciferase assay buffer (2 mM ATP in lysis buffer) and luciferase activity was assayed on a FLUOstar OPTIMA (BMG LABTECH, Offenburg, Germany) luminescence microplate reader. The reaction was initiated by the injection of 50 µL of 200 µM luciferin solution. Luciferase assay values were normalized to β-galactosidase assay results (a measure of transfection efficiency). For β-galactosidase assay, cell lysates were incubated in 85 µL of 0.2 mg/mL of CPRG in 60 mM Na_2_HPO_4_, (pH 8.0), 10 mM KCl, 1 mM MgCl_2_, and 1 mM DTT, for 5 to 10 minutes, and monitored at 580 nm. Transfection experiments were reproduced at least three times and are presented as means ± SEM.

### Western Blotting and Co-Immunoprecipitation (Co-IP)

Harvested cells were lysed in 50 to 100 µL of cell lysis buffer (20 mM HEPES pH 7.9), 420 mM NaCl, 1.5 mM MgCl_2_, 0.2 mM EDTA and 25% glycerol, 1 mM DTT and 0.5 mM PMSF) and an equal amount of total protein (determined using BioRad protein assay and diluted 1∶1 with 2×Laemmli loading buffer) was loaded on a 12% SDS-PAGE, run at 120 V for 1.5 to 2 hours and transferred onto Immobilon PVDF membrane (Millipore, Bedford, MA) overnight at 4°C. Membranes were probed with 5% milk in Tris-Buffered Saline, Tween-20 (TBST) for 1 hour. Western blot analysis for Nrf1 was performed using mouse anti-human Nrf1 antibody (1∶1,000 dilution), and horseradish peroxidase-labelled anti-mouse IgG secondary antibody (1∶2,000 dilution; DAKOCytomation, Mississauga, ON). FLAG- tagged Nrf1 was visualized using horseradish peroxidase (HRP)-conjugated mouse anti-FLAG M2 antibodies (1∶4,000 dilution; Sigma-Aldrich). As a loading control, membranes were probed with mouse anti-human β-tubulin (1∶4,000 dilution, Developmental Studies Hybridoma Bank, Iowa City, IA) or stained with Ponceau S red. Blots were developed by enhanced chemiluminescence substrate (Millipore, Bedford, MA) and Kodak X-Omat blue film (Perkin-Elmer, Waltham, MA). Film was scanned using a CanoScan LIDE 80 scanner (Canon, Lake Success, NY) and band densitometry was measured using AlphaEaseFC software, version 3.1.2 (Alpha Innotech/Cell Biosciences, Santa Clara, CA). For co-IP, the protein complexes were immunoprecipitated from 125 µg total lysate protein with 20 µL Protein A/G-Sepharose beads and 2 µg anti-Nrf1 antibody. The immunoprecipitated complexes were subjected immunobloting using anti-ubiquitin antibody.

### Electrophoretic Mobility-Shift Assay (EMSA)

Cells were lysed as described above and 20 µg total protein was reacted with a biotinylated dsDNA EpRE probe of the human glutamate-cysteine ligase, modifier subunit (*gclm*) promoter [44] and was subjected to non-denaturing electrophoresis according to the manufacturer's instructions (Panomics, Fremont, CA). Protein-DNA complexes were transferred onto an Amersham Hybond-N^+^ membrane (GE Healthcare, Buckinghamshire, UK) and visualized using streptavidin-HRP and chemiluminescence as described above for Western blots.

### Bioinformatic Analyses

ClustalW tool [45] was used for multiple amino acid alignment http://www.ebi.ac.uk/Tools/clustalw2/index.html). Nrf1 PEST domain was identified using PESTfind algorythm that was previously located at https://emb1.bcc.univie.ac.at/toolbox/pestfind/pestfind-analysis-webtool.htm. The prediction of phosphorylation sites was performed using NetPhos 2.0 server available at http://www.cbs.dtu.dk/services/NetPhos/ [46]. The prediction of kinase-specific phorphorylation sites [47] was performed with http://www.cbs.dtu.dk/services/NetPhosK/.

### Statistical Analyses

Data are presented as means ± SEM of at least three independent experiments. The results were considered statistically significant at p<0.05 for the Student's paired t-test.

## Supporting Information

Figure S1 Oxidative stressor AAPH induces Nrf1 DNA binding and stabilizes p95 Nrf1 form independently of p120. (**A**) The Nrf1-specific band on an EMSA format was determined by including 20 µg of the COS7 cell lysate with: 1) no antibody; 2) anti-actin antibody; 3) anti-Nrf1 antibody; 4) anti-Nrf1 antibody, no lysate; and 5) neither lysate nor antibody (probe only). The experiment was performed twice with the same outcome. (**B**) COS7 cells were treated with 80 mM AAPH for 6 hours [48], [49] and the lysates were subjected to EMSA and Western blotting (**C**). The position of AAPH-inducible Nrf1 semi-specific bands is shown with arrows in **A** and **B**. In **C**, both short- and long-time exposure of bands are shown for better clarity.
(TIF)

Figure S2 Oxidative stressors AAPH and tBHQ, hypoxia and CHX destabilize the p250 form of Nrf1. COS7 cells were treated for six hours with (**A**) 80 mM AAPH, 100 (+) or 200 (++) µM tBHP or hypoxia for twenty-four hours or with (**B**) 100 µg/mL CHX [50] for the times indicated, after which cells were harvested and total cell lysates were subjected to immunoblotting with anti-Nrf1 or anti-β-tubulin antibodies. Molecular masses are indicated in kDa. The results of two independent experiments (**A**) or a representative result of three independent experiments (**B**) are shown. Nrf1 forms (p65, p95 and p250) are indicated with arrows.
(TIF)
