# Supplementary figures and images for: The Nrf1 CNC-bZIP Protein Is Regulated by the Proteasome and Activated by Hypoxia
Source: PLoS One. 2011 Dec 21;6(12):e29167. doi: 10.1371/journal.pone.0029167 (PMC3244438; doi:10.1371/journal.pone.0029167)

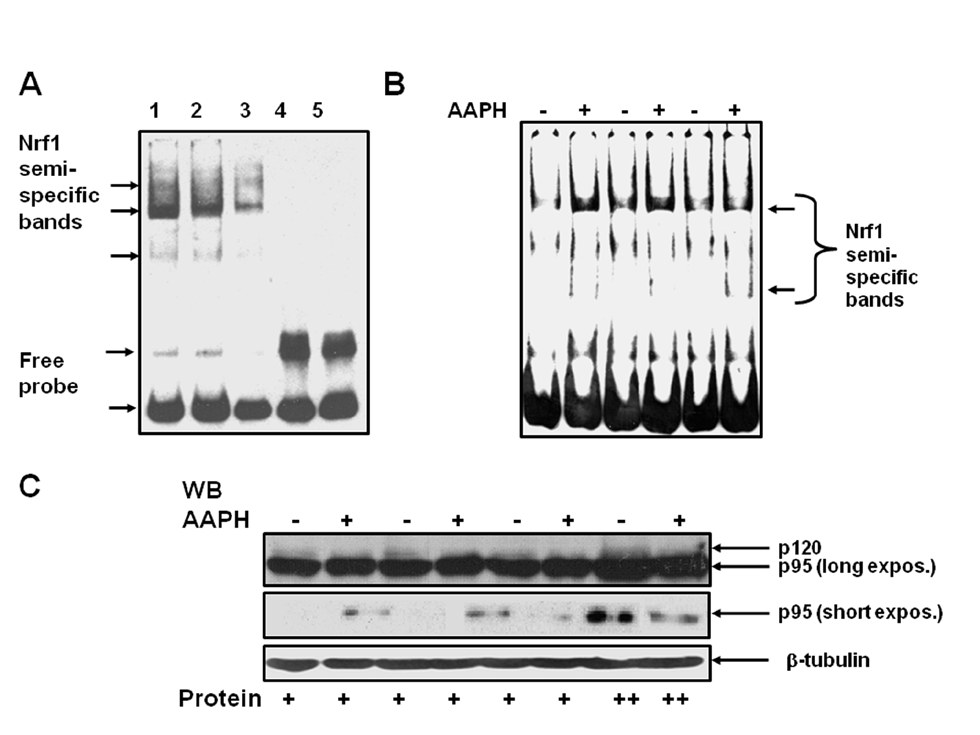

Supplement: Figure S1 — Oxidative stressor AAPH induces Nrf1 DNA binding and stabilizes p95 Nrf1 form independently of p120. (A) The Nrf1-specific band on an EMSA format was determined by including 20 µg of the COS7 cell lysate with: 1) no antibody; 2) anti-actin antibody; 3) anti-Nrf1 antibody; 4) anti-Nrf1 antibody, no lysate; and 5) neither lysate nor antibody (probe only). The experiment was performed twice with the same outcome. (B) COS7 cells were treated with 80 mM AAPH for 6 hours [48], [49] and the lysates were subjected to EMSA and Western blotting (C). The position of AAPH-inducible Nrf1 semi-specific bands is shown with arrows in A and B. In C, both short- and long-time exposure of bands are shown for better clarity. (TIF) [file pone.0029167.s001.tif]

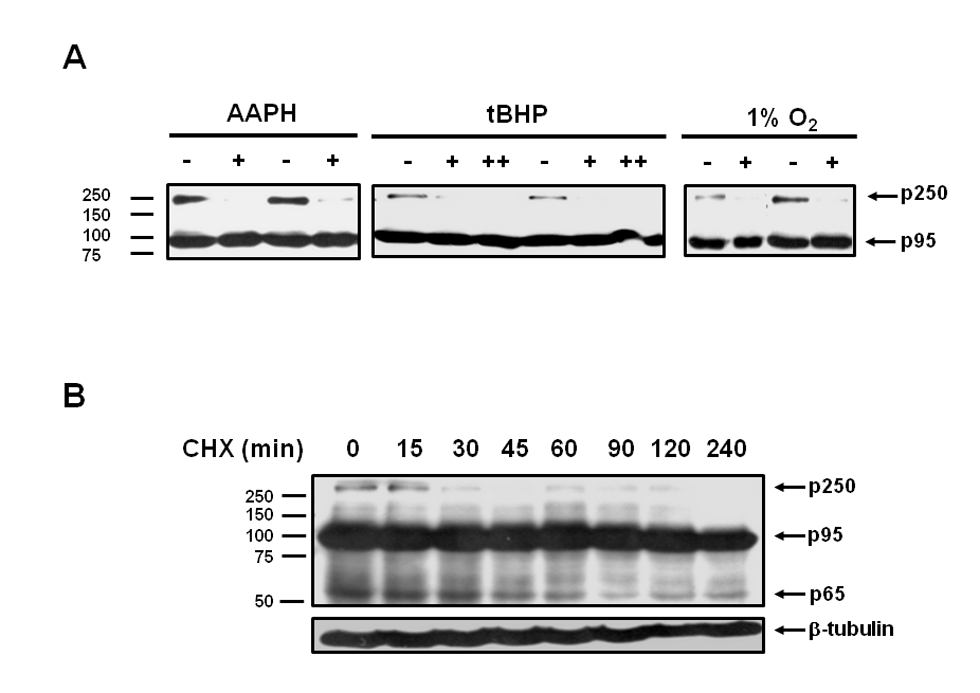

Supplement: Figure S2 — Oxidative stressors AAPH and tBHQ, hypoxia and CHX destabilize the p250 form of Nrf1. COS7 cells were treated for six hours with (A) 80 mM AAPH, 100 (+) or 200 (++) µM tBHP or hypoxia for twenty-four hours or with (B) 100 µg/mL CHX [50] for the times indicated, after which cells were harvested and total cell lysates were subjected to immunoblotting with anti-Nrf1 or anti-β-tubulin antibodies. Molecular masses are indicated in kDa. The results of two independent experiments (A) or a representative result of three independent experiments (B) are shown. Nrf1 forms (p65, p95 and p250) are indicated with arrows. (TIF) [file pone.0029167.s002.tif]
